# Back to Basics: Endotracheal Tube Too Deep, Too Shallow, Then Just Right

**DOI:** 10.7759/cureus.2706

**Published:** 2018-05-29

**Authors:** Latha Ganti, John Shivdat, Sheila Bawany

**Affiliations:** 1 Clinical Sciences, University of Central Florida College of Medicine, Orlando, USA; 2 Emergency Medicine, Mercer University School of Medicine, Macon, USA; 3 Emergency Medicine, University of Central Florida College of Medicine Hca Gme Consortium, Orlando, USA

**Keywords:** endotracheal tube

## Abstract

We present a case of a 62-year-old who required intubation for the increased work of breathing secondary to a chronic obstructive pulmonary disease (COPD) exacerbation. The case illustrates the correct positioning of the endotracheal tube, as verified radiographically. It clearly depicts the tube that is initially advanced too far, then pulled back too much, and is finally in the correct position.

## Introduction

Endotracheal intubation is a commonly performed life-saving emergency department (ED) procedure. It is most often performed in conjunction with rapid sequence intubation (RSI), a combination of a paralytic followed by a sedative agent, such as succinylcholine and etomidate [1]. RSI success depends on a number of factors, commonly referred to as the “7 Ps” [2]: Preparation, Preoxygenation, Preintubation optimization, Paralysis with induction, Positioning, Placement with proof, and Postintubation management. The authors present a case where endotracheal intubation was not a problem, but rather the position of the tube, as verified by chest radiography. Indeed, in a large, multicenter national registry of ED intubations of over 17,000 patients, mainstem intubation, where the tube is advanced too far, complicated 1.2% of all emergency department intubations [3].

## Case presentation

A 62-year-old male with chronic obstructive pulmonary disease (COPD) exacerbation presented to the emergency department with a fever of 100.8°F, tachypnea (22 breaths/minute), and room air hypoxia (90%). He was intubated due to his increased work of breathing. A 7.5 mm endotracheal tube (ETT) was easily placed using 20 mg of etomidate intravenous (IV) and 100 mg of succinylcholine IV for rapid sequence intubation. Placement in the trachea was verified with a color change from purple to gold on a colorimetric CO_2_ detector and the visualization of ETT passage through the cords. The patient had bilateral breath sounds upon post-intubation auscultation.

**Figure 1:**
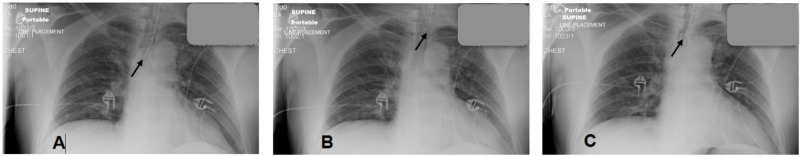
CXR panel demonstrating the position of the endotracheal tube CXR: chest radiography

The depth of the ETT was verified via chest radiography (CXR). Initially, the endotracheal tube was advanced too far (Figure 1A). The tube was then pulled back (Figure 1B), but too much so, with the tip of the tube at about the T3 level. Finally, the tube was advanced to its ideal position (Figure 1C) approximately 5 cm above the carina, which corresponds to T5-T7, visually seen on CXR between the clavicles. The patient received intravenous antibiotics and steroids, was admitted to the ICU, and discharged on day five without complications.

## Discussion

This case illustrates the importance of verifying endotracheal tube placement. While common teaching is to place the tube at 23 cm at the lip for males, given individual anatomy variants [4], this will not always result in ideal tube depth. Thus, radiographic confirmation is important. Indeed, an emergency department study of more than 380 patients concluded that while ED intubations have high success rates, the complications of inappropriate intubations are high stakes enough that post-intubation CXR remains a necessary step to minimize the incorrect placement of the tube [5]. A similar study performed in the intensive care unit (ICU) on over 200 patients concluded the same [6].

While intubation is an important procedure in the emergency room and in critically ill patients in any setting, the procedure itself is not without risks and complications. Failure to achieve adequate ventilation and oxygenation, resulting in hypoxia, may occur during prolonged attempts at intubation. Prolonged pharyngeal stimulation can also precipitate cardiac decompensation and laryngeal or bronchial spasms [7]. Additionally, undetected esophageal intubations may result in hypoxia.

Another complication includes right mainstem intubation, which can manifest as hypoxia, atelectasis, or pneumothorax. Longstanding physiological sequelae of endobronchial intubation include barotrauma secondary to the ventilation of a solitary lung with higher pressures than normal [8].

A penetrating injury after using a stylet during intubation or the overinflation of the cuff of the endotracheal tube under rapid sequence intubation can result in tracheal rupture [9].

Techniques to ensure tracheal placement include the visualization of the tube passing through the vocal cords, auscultation for bilateral breath sounds, and end-tidal CO_2_ on capnography [10]. Chest radiography is good for assessing the proper depth of insertion, but it does not necessarily distinguish between tracheal and esophageal placement.

The depth of the ETT is traditionally taught as 23 cm at the lip for men and 21 cm for women. This is based on a study of 83 Caucasian patients [11]. However, the depth of the ETT is best based on the height of the patient [4] and has little to do with race or gender, except that, in general, men tend to be taller than women.

## Conclusions

Endotracheal intubation is a critical life-saving procedure that is routinely performed in the emergency department. While success rates are high, the position of the tube within the trachea is still important to verify. This case illustrates the correct positioning of the endotracheal tube, as ascertained radiographically.
